# Abnormal Cingulum Bundle Induced by Type 2 Diabetes Mellitus: A Diffusion Tensor Tractography Study

**DOI:** 10.3389/fnagi.2020.594198

**Published:** 2020-12-11

**Authors:** Ying Cui, Tian-Yu Tang, Chun-Qiang Lu, Yu Cai, Tong Lu, Yuan-Cheng Wang, Gao-Jun Teng, Shenghong Ju

**Affiliations:** ^1^Department of Radiology, Zhongda Hospital, School of Medicine, Southeast University, Nanjing, China; ^2^Jiangsu Key Laboratory of Molecular and Functional Imaging, Department of Radiology, Zhongda Hospital, School of Medicine, Southeast University, Nanjing, China

**Keywords:** Type 2 diabetes mellitus, cognitive impairment, cingulum bundle, diffusion tensor imaging, tractography, insulin resistance

## Abstract

**Purpose**: In Type 2 diabetes (T2DM), white matter (WM) pathology has been suggested to play an important role in the etiology of T2DM-related cognitive impairment. This study aims to investigate the integrity of the cingulum bundle (CB), a major WM tract, in T2DM patients using diffusion tensor tractography.

**Methods**: Thirty-seven T2DM patients and 34 age-, sex- and education matched healthy controls were included and underwent diffusion tensor imaging. Tractography of bilateral CB tracts was performed and diffusion measurements were compared between the two groups. Next, brain regions with significant group differences on fractional anisotropy (FA) values were set as the region of interest (ROI), and the CB fibers that passed through were identified. Diffusion measures were extracted from these fibers to investigate their correlations with the cognitive performances and endocrine parameters.

**Results**: T2DM patients exhibited decreased FA in bilateral CB, increased mean diffusion (MD) in the right CB, and decreased length in the left CB. Through voxel-wise comparison, the most prominent FA difference was identified in the posterior segment of the CB and the reconstructed tract was part of the retrosplenial component. Importantly, the diffusion measurements of the tract were significantly correlated with the impaired performance in executive functioning and elevated insulin resistance (IR) in the T2DM group, instead of the control group.

**Conclusions**: The diffusion measurements in bilateral CB were altered in T2DM patients, which might reflect important neuropathologic changes in the fibers. Our study adds to knowledge about how the cingulum changes structurally along its entire length in T2DM and highlights the relationship between WM and cognitive performance. Besides, IR might be an important risk factor that warrants further exploration.

## Introduction

Type 2 diabetes (T2DM) is a chronic metabolic disorder associated with a series of multi-systemic complications (American Diabetes Association, 2020). In recent years, impairment in cognitive functioning has been increasingly recognized in diabetic patients, the risk of dementia in which is about two times higher than the controls (Xia et al., 2020). Although the etiology remains controversial due to the complex comorbidities interplay, growing evidence suggests that vascular dysfunction, including both microvascular and macrovascular, might be an important risk factor (Reijmer et al., 2013). As revealed by brain imaging studies, hyperglycemia, and insulin resistance (IR) are related to increased white matter (WM) lesions and impaired WM connectivity (You et al., 2018). Therefore, WM pathology is suggested to play an important role in the etiology of T2DM-related cognitive impairment.

The cingulum bundle (CB) is a collection of fiber tracts longitudinally in the WM, interconnecting prefrontal, parietal, and medial temporal regions (Bubb et al., 2018). Given that these are core regions that have been repeatedly implicated in diverse processes, the CB is suggested to play a critical role in cognitive functioning, such as emotion and execution of motor- and attention-related tasks (Metzler-Baddeley et al., 2012; Bubb et al., 2018). Microstructural abnormalities in CB have been demonstrated in various conditions, including mild cognitive impairment (MCI), Alzheimer’s disease (AD), depression, traumatic brain injury, and several other neuropsychological diseases (Kamagata et al., 2012; Metzler-Baddeley et al., 2012; Jang et al., 2013; Taylor et al., 2014). In T2DM patients, disruptions in major WM tracts have been consistently reported, which are possibly attributed to the increased vascular risk factors (Pruzin et al., 2018). Also, we and several other studies have reported disconnection in the default mode network (DMN), a key network to maintain the normal cognitive activities and its main components are linked by CB (van den Heuvel et al., 2008; Cui et al., 2015). Taken together, the critical connections and function of the CB prompted us to further characterize its profile and to elucidate the relationship between CB abnormality and the cognitive decline in T2DM patients.

DTI is a widely applied MR technique that is especially superior in imaging interconnecting WM tracts (Sanjari Moghaddam et al., 2019). It allows for the visualization of individual fiber bundles and quantification of its diffusion metrics, leading to a wide application in neurodegenerative diseases. Alteration in DTI indices is often independent of small vessel disease (SVD) revealed by conventional MRI, i.e., white matter hyperintensity (WMH) and lacunar infarcts, and precede the clinical symptoms (Reijmer et al., 2013). Altogether, DTI appears to be complementary to the classical MRI markers in detecting subtle WM changes at the very early stage of cognitive impairment. According to a recent review, most of the current T2DM-related DTI studies have surveyed whole-brain WM diffusion metrics using either voxel-based analyses (VBA) or tract-based spatial statistics (TBSS; Sanjari Moghaddam et al., 2019). Only one study specifically focused on the CB integrity, in which the tract was reconstructed using a region of interest (ROI) based approach, and only basic DTI measures including fractional anisotropy (FA) and mean diffusion (MD) were computed (Hoogenboom et al., 2014). A more detailed picture of the CB, including its volume, length, and density, and their correlation between cognitive and endocrine parameters, remains to be unraveled. The recently developed automatic streamline fiber tracking uses a track recognition based on a tractography atlas, with an additional algorithm to filter out false and unrelated tracks (Zhang et al., 2010; Yeh et al., 2019). It avoids the anatomical misplacement caused by manual tracing of apriori ROIs, thus providing better precision and quantification for the reconstruction.

In the current study, we aim to: (1) characterize the DTI indices of the CB in T2DM patients using the automatic streamline tractography, and compare them with healthy controls, (2) locate the most significant differences on the CB, and (3) explore the correlation between cingulum abnormalities with cognitive functioning and glycemic parameters. Our results would be promising in investigating the role of major WM tract disruption in the development of T2DM-related cognitive decline.

## Materials and Methods

### Subjects

The study was approved by the institutional review board, and informed consent was obtained from all participants before evaluation. Participants were enrolled from the Department of Endocrinology or the local community through advertisement. All subjects were between 50 and 75 years old, with a minimum education of 6 years, and were group-matched in terms of age, sex, and education. Participants who met the following criteria were excluded: score on the Mini-Mental State Examination (MMSE) <24, score on the Hamilton Depression Rating Scale (HAM-D) ≥7, history of brain lesions such as tumor or stroke, and unrelated psychiatric or neurological disorder and MRI contraindications.

Diagnosis of T2DM was based on the American Diabetes Association criteria. All patients had a disease duration of at least 1 year and close self-monitoring. Neither a history of hypoglycemic episodes nor clinically detectable complications such as retinopathy, nephropathy, and peripheral neuropathy were reported. To exclude subjects in the pre-diabetes state, all healthy controls were performed with an oral glucose tolerance test (OGTT; 75 g dextrose monohydrate in 250 ml water). Subjects having a fasting blood glucose ≥7.0 mmol/L or postprandial glucose ≥7.8 mmol/L after 2 h OGTT were excluded.

### Clinical Data Collection

A detailed questionnaire was used to collect information on medical histories and medication use. Weight, height, and waist circumferences were carefully recorded. Blood pressure was measured three times during the visit and averaged. Hypertension was defined as previously described. Blood samples were collected *via* venipuncture at 7:00 AM after overnight fasting to measure the fasting plasma glucose (FPG), glycosylated hemoglobin (HbA1c), fasting insulin, and cholesterol levels (i.e., triglyceride, total cholesterol, LDL cholesterol, and HDL cholesterol). Subsequently, blood samples were collected again at 9:00 AM, 2 h after OGTT for controls and a usual meal for patients, to measure the postprandial glucose. The homeostasis model assessment of insulin resistance (HOMA-IR) was used to assess the degree of IR for all subjects except for those patients treated with insulin. This is because HOMA-IR may not accurately reflect insulin sensitivity in patients who require insulin or who have a minimal b-cell function.

### Neuropsychological Tests

The general neuropsychological status of the participants was tested using MMSE. Subjects with a score below 24 were considered dementia and subsequently excluded, as described in the exclusion criteria. Tests that covered multiple domains were also performed, including the Auditory Verbal Learning Test (AVLT) for episodic memory, Complex Figure Test (CFT)-copy trial and Clock Drawing Test (CDT) for spatial processing ability, CFT-delay trail for spatial memory, Digit Span Test (DST) for working memory, Tail-Making Test (TMT) part A for attention and part B for executive functioning. All tests were performed in a fixed order by two experienced neurologists, which took about 60 min to complete.

### MR Data Acquisition

Brain MRI data were acquired using a 3.0T MR-scanner (Siemens MAGNETOM Trio, Erlangen, Germany) with a 32-channel head coil. Foam padding and earplugs were used to reduce head motion and scanner noise. To obtain an anatomical reference, high-resolution T1-weighted imaging with a magnetization-prepared rapid gradient echo (MPRAGE) sequence was performed with a repetition time (TR)/echo time (TE) of 1,900/2.48 ms, a flip angle of 9°, an acquisition matrix of 256 × 256, a field of view (FOV) of 250 × 250 mm, and a slice thickness of 1 mm. DTI images were acquired by using the spin-echo echo-planar imaging (EPI) sequence (TR/TE = 10,000/95 ms, matrix size = 128 × 128, FOV = 256 × 256 mm^2^, section thickness = 2 mm, 70 slices, 6/8 partial Fourier, NEX = 2, 30 gradient directions with a *b*-value of 1,000 s/mm^2^ and one *b* = 0 s/mm^2^ image. FLAIR images were also obtained for WMH evaluation: TR = 8,500 ms, TE = 94 ms, slice = 20, slice thickness = 5 mm, with each voxel size of 1.3 × 0.9 × 5 mm^3^.

### Evaluation of Small Vessel Disease

WMH and lacunar infarcts were assessed on fluid-attenuated inversion recovery images with a method described previously (Wahlund et al., 2001). To minimize the effects of WMH on fiber tracts, participants with a rating score >1 (confluence of lesions or diffuse involvement of each region) were excluded. The assessment was performed by two experienced radiologists blinded to the group allocations and a consensus was obtained through discussion between the raters.

To obtain the distribution of WMH and minimize its confounding effects on cingulum, FLAIR images were auto-segmented and binarized to obtain a WMH image using the Lesion Segmentation Toolbox version 3.0.0 implemented in SPM^1^. After normalizing into the MNI space, each individual’s binarized WMH image was overlaid and divided by the number of subjects in each group to generate a probabilistic map in percentage. The voxel intensity value of the map indicates the frequency of WMH at each anatomical location. Therefore, the probabilistic map serves as a measure of inter-subject variability and WMH anatomical distribution. The overlap between the WMH and cingulum was assessed through visual inspection, and the probabilistic maps were compared between the T2DM and control groups to evaluate if there is a difference in WMH distribution.

### Image Preprocessing and CB Tractography

The Diffusion MRI reconstruction and WM tractography were performed with DSI Studio software^2^. First, eddy current distortion correction was performed using the FSL toolbox^3^. To ensure the data accuracy, an automatic quality control routine was performed including b-table checking, calculation of the mean Pearson correlation coefficient of the neighboring diffusion-weighted images, and identification of slice-wise signal dropout for each slice in each diffusion-weighted image (Schilling et al., 2019). To obtain the spin distribution function, the diffusion images were reconstructed in the MNI space using *q*-space diffeomorphic reconstruction, as described previously (Yeh and Tseng, 2011). The diffusion sampling length ratio was set at 1.25 and the average dataset was resampled to 2-mm isotropic resolution. The restricted diffusion was quantified using restricted diffusion imaging (Yeh et al., 2017).

Subsequently, automatic fiber tracking based on atlas-guided track recognition was performed with DSI studio software and false tracks and unrelated tracks were filtered out based on a previously reported atlas (Yeh et al., 2018). The streamline tractography was generated using the following parameters: seeds = 1,000,000, anisotropy threshold = 0.1, angular threshold = 60°, step size = 0.5 mm. The fiber trajectories were then smoothed by averaging the propagation direction with 80% of the previous direction. Fibers shorter than 30 mm or longer than 250 mm were discarded. Finally, topology-informed pruning was applied to further remove the false connections (Yeh et al., 2019).

After obtaining the reconstructed data, the left and right CB were identified according to the HCP tractography atlas (Yeh et al., 2018). To quantify the microstructural abnormalities, we computed several DTI measures, including the number, mean length, volume, surface area, FA, and MD values of the tracks, which were included in the group comparison.

### Voxel-Based Analysis and ROI-Based Tractography

To further identify the most prominently affected regions in the impaired CB, we performed a voxel-based group comparison on the FA maps. The *P*_uncorrected_ value was set at 0.005, with a cluster size of 26 voxels (determined *via* Monte Carlo simulation) corresponding to a *P*_corrected_ of 0.05. The identified clusters with significant FA differences were then set as the ROIs and the CB fibers that passed through were retained. Diffusion measures of the fibers were then extracted to investigate their group differences and correlations with the clinical parameters.

### Statistical Analysis

Demographic variables and cognitive performance were compared between the two groups using SPSS software (Version 21.0, SPSS Inc., Chicago, IL, USA). Normal distributions were tested using the Kolmogorov–Smirnov test. An independent two-sample *t*-test was used for continuous variables, a nonparametric Mann–Whitney *U* test, for asymmetrically distributed variables, and a χ^2^ test, for proportions. *P*-values <0.05 were considered statistically significant.

Diffusion values of bilateral CB were compared between groups using multivariate ANCOVA, controlling for age, gender, education level, and WMH volume to minimize their confounding effects. Given that the CB has distinct segments and their according functions, we assumed that to take the CB as a whole would overlook/underestimate its correlation with the clinical parameters. Therefore, the correlations among DTI measures, cognitive performance, and endocrine metrics (plasma glucose and HbA1c levels, HOMA-IR, and disease duration) were performed based on the tract reconstructed to form the ROIs with group difference. Partial Pearson correlation analyses were performed, adjusted for the same covariates as those controlled in the two-sample *t*-tests. A *P-*value <0.05 was considered statistically significant.

## Results

### Demographic and Neuropsychological Results

A total of 40 T2DM patients and 43 HCs were recruited. Three HCs quitted the cognitive assessment and two HCs refused the blood collection. Two patients and one HC were excluded due to extensive WM lesions. One patient and three HCs were further excluded after the quality control routines for DTI images. Therefore, 37 T2DM patients and 34 healthy controls were included in the final analyses. Clinical and demographic characteristics for the diabetic and control groups are summarized in Table 1. No significant differences were found between groups in terms of age, education, gender distribution, WMH load, blood lipid, and blood pressure. Although BMI is higher in the diabetic group, the difference did not reach statistical significance. As expected, fasting glucose, postprandial glucose, HbA1c, and IR were all significantly elevated in the patients’ group.

**Table 1:** Demographics and clinical variables of the study sample.

Measures	T2DM (*n* = 37)	Control (*n* = 34)	*P*-value
Age (years)	59.7 ± 7.3	56.9 ± 6.1	0.08
Sex (male/female)^a^	19/18	12/22	0.23
Education (years)	9.9 ± 3.3	10.4 ± 2.4	0.49
Diabetes duration (years)	8.2 ± 4.5	-	-
Insulin treatment (n)	10	-	-
HbA1c (%, mmol/mol)	7.8 ± 1.5	5.6 ± 0.3	<0.001*
FPG (mmol/L)	7.7 ± 2.2	5.5 ± 0.4	<0.001*
Postprandial glucose	15.1 ± 4.8	6.4 ± 1.8	<0.001*
HOMA-IR	3.5 ± 2.0	2.6 ± 1.4	0.03*
BMI (kg/m^2^)	24.5 ± 3.1	23.4 ± 2.8	0.15
Systolic BP (mmHg)	135.7 ± 14.8	129.8 ± 12.4	0.07
Diastolic BP (mmHg)	85.0 ± 10.9	85.6 ± 11.5	0.85
Total cholesterol (mmol/L)	5.3 ± 1.2	5.0 ± 0.8	0.64
Triglyceride (mmol/L)	1.5 ± 0.8	1.3 ± 0.7	0.37
HDL cholesterol (mmol/L)	1.3 ± 0.3	1.3 ± 0.3	0.55
LDL cholesterol (mmol/L)	3.2 ± 0.8	3.1 ± 0.6	0.44
White matter lesions (range)	0–6	0–7	0.35
Lacunar infarcts (n)^a^	7	4	0.52

Data are represented as mean ± (SD), n, or range. ^a^Statistical analyses were performed by χ^2^ test. *P-value < 0.05. FPG, fasting plasma glucose; HOMA-IR, homeostasis model assessment of insulin resistance; BMI, body mass index; BP, blood pressure. Significant values in bold.

Cognitive results are summarized in Table 2. Both groups performed within the normal range on MMSE, but the diabetic group scored lower than the control group on the CFT-Delay trial and TMT-part B. These tests covered cognitive domains including memory, information processing, and executive functioning, which are all frequently reported to be impaired in diabetic subjects. Notably, disease duration was exclusively correlated with the score on CFT-Delay (*R* = −0.422, *P* = 0.009). No additional correlations were found among other clinical variables.

**Table 2:** Cognitive performance of Type 2 diabetes (T2DM) and control groups.

Measures	T2DM (*n* = 37)	Control (*n* = 34)	*P*-value
MMSE	28.4 ± 1.2	28.9 ± 1.1	0.11
AVLT-copy	6.1 ± 1.2	6.2 ± 1.4	0.84
AVLT-delay	6.0 ± 2.3	6.2 ± 2.3	0.78
CFT-copy	34.5 ± 1.7	35.0 ± 1.4	0.22
CFT-delay	14.3 ± 5.9	17.7 ± 6.0	0.02*
DST (forward)	7.0 ± 1.2	7.4 ± 1.6	0.21
DST (backward)	4.2 ± 0.9	4.6 ± 1.5	0.15
TMT-part A	65.6 ± 21.5	61.8 ± 14.8	0.40
TMT-part B	176.2 ± 61.4	147.8 ± 45.3	0.03*
CDT	3.3 ± 0.6	3.5 ± 0.5	0.17

Data are represented as mean ± (SD). *P-value < 0.05. MMSE, mini-mental state examination; AVLT, auditory verbal learning test; CFT, complex figure test; DST, digit span test; TMT, trail-making test; CDT, clock drawing test. Significant values in bold.

### Distribution of WMH

**Figure 1:**
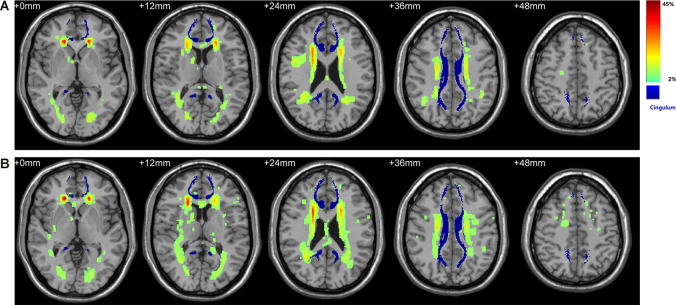
Spatial distribution of WMH and its overlay with bilateral cingulum. **(A)** WMH distribution in the control group. **(B)** WMH distribution in the Type 2 diabetes (T2DM) group. Color denotes the frequency of WMH at each anatomical location, serving as a measure of inter-subject variability in each group. The WHM distribution has a predilection for periventricular and deep WM areas, especially in frontal periventricular regions (red). Bilateral CB shown in blue was derived from the fiber template HCP1021-1mm (https://pitt.app.box.com/v/HCP1021-1mm). WMH, white matter hyperintensity; WM, white matter; CB, cingulum bundle.

The color-coded composite probability map in MNI anatomic space for all participants is shown in Figure 1. Visual inspection of the map suggested a predominant WMH distribution for periventricular and deep WM areas, especially in frontal periventricular regions (color in red). The blue color displays the complete CB derived from the fiber template HCP1021-1mm^4^, which is largely independent of the surrounding WMH. Comparison of the probability map between the T2DM and HC groups also suggested a similar distribution, with the most prominent WMH in identical regions. Taken together, the above results demonstrated the little effect of WMH on the integrity of the CB.

### Tractography of the Cingulum

**Figure 2:**
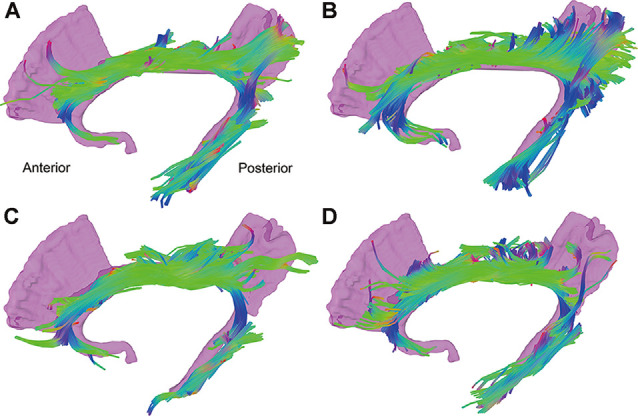
Tractography results of the CB of four representative subjects. The reconstructed CB is a symmetrical sickle-shaped bundle. The fibers in the CB in diabetic representatives **(C,D)** were slender and more diffusely orientated compared with the healthy controls **(A,B)**. CB, cingulum bundle; CC, corpus callosum.

Figure 2 shows the reconstructed CB of four representative subjects. As a symmetrical sickle-shaped bundle that encircled the CC, the shape and trajectory of CB are highly consistent with the WM template and previous literature. However, the frontal fibers were less than the template, which might be attributed to normal aging (Sibilia et al., 2017). Through visual inspection, the fibers in the CB in diabetic representatives were slender and more diffusely orientated compared with the healthy controls.

### Between-Group Differences of DTI Metrics

**Figure 3:**
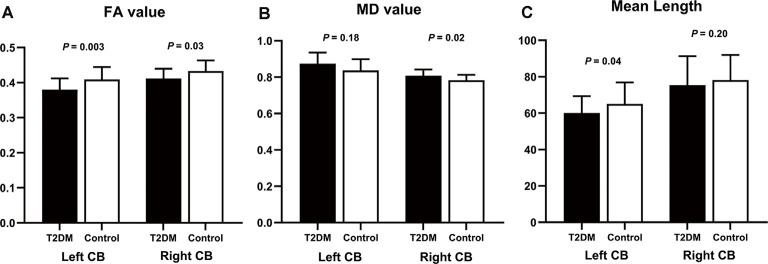
Group differences of diffusion measurements in bilateral CB. The comparison used age, sex, education, and WMH as covariates. **(A)** In the T2DM group, FA values were significantly decreased in bilateral CB. **(B)** MD values were higher in the T2DM group, but the significance in the left CB did not remain after controlling for the covariates. **(C)** T2DM patients had shorter fiber length compared to the controls, but only the difference in the left CB was significant after introducing the covariates. CB, cingulum bundle; FA, fractional anisotropy; MD, mean diffusivity. The error bars represent the standard deviation.

The DTI results are summarized in Table 3 and Figure 3. After controlling for age, gender, education level, and WMH scores, we observed a significant between-group effect for the DTI metrics of bilateral CB tracts. Patients exhibited decreased FA (*P* = 0.030) and increased MD (*P* = 0.024) in the right CB. Meanwhile, lower FA (*P* = 0.003) and shorter fiber length (*P* = 0.042) in the left CB were also identified in the diabetic group. Although patients also showed higher MD in the left CB (*P* = 0.016), the difference was insignificant after introducing the covariates (*P* = 0.180). Of further note, none of the tract-number, volume, and surface areas were different between the groups, but there was a trend of fewer fibers and smaller tracts in both bilateral CB in the diabetic group.

**Table 3:** Diffusion measures of bilateral cingulum bundle in both groups.

	Measures	T2DM (*n* = 37)	Control (*n* = 34)	*P*-value
Left CB	Tract number	2,331.7 ± 1,259.3	2,362.4 ± 1,151.8	0.74
	Mean length	60.1 ± 9.3	65.1 ± 11.8	0.04*
	Volume	12,695.6 ± 4,461.9	13,371.6 ± 3,942.4	0.37
	Surface area	8,186.7 ± 2,067.7	8,551.2 ± 1,847.4	0.31
	FA	0.38 ± 0.03	0.41 ± 0.04	0.003*
	MD	0.87 ± 0.62	0.84 ± 0.61	0.18
Right CB	Tract number	1,944.8 ± 1,271.0	1,988.6 ± 986.0	0.87
	Mean length	75.4 ± 15.9	78.2 ± 13.7	0.23
	Volume	12,190.3 ± 5,389.4	12,873.2 ± 4,013.4	0.15
	Surface area	7,847.2 ± 2,473.9	8,246.9 ± 1,930.6	0.13
	FA	0.41 ± 0.28	0.43 ± 0.30	0.03*
	MD	0.81 ± 0.03	0.78 ± 0.03	0.02*

Data are represented as mean ± (SD). *P-value <0.05. The comparison was performed after controlling for age, sex, education, and WMH. CB, cingulum bundle; FA, fractional anisotropy; MD, mean diffusion. Significant values in bold.

**Figure 4:**
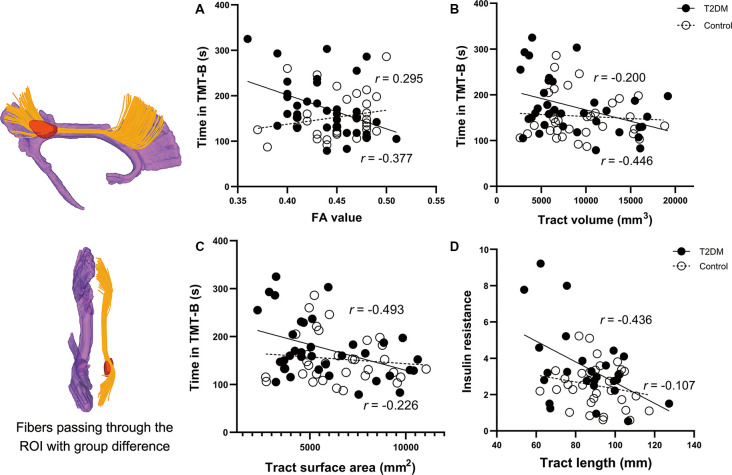
Correlations between the clinical parameters and the diffusion measures of the reconstructed tract using ROI-based tractography. Figures on the left are the results of the voxel-wise comparison of FA maps between the two groups. Significantly decreased FA was found in the posterior part of the right cingulum (the region in red). The tract passing through the ROI is illustrated in yellow. Correlations in the T2DM group are presented in black circles, while correlations in the control group are presented in white circles. **(A–C)** In the T2DM group, the time spent in TMT-B was negatively correlated with FA, volume, and surface area of the tract. **(D)** IR in the T2DM group was negatively correlated with the mean length of the reconstructed tract. No such correlations were observed in the control group. The CB shown in purple was derived from the template HCP1021-1mm (https://pitt.app.box.com/v/HCP1021-1mm). CFT-delay, complex figure test-delay trial; TMT-B, trail making test-part B; IR, insulin resistance; FA, fractional anisotropy; ROI, the region of interest.

The VBA revealed that the most prominent group difference was located in the posterior part of the right CB, as shown in Figure 4 (color in red). The reconstructed tract is shown in yellow, which belongs to the dorsal segment of the CB, connecting the medial frontal and parietal lobes along the cingulate cortices. Nevertheless, all the diffusion measurements of the tract did not differ between the two groups.

### Correlational Analyses

Correlational results are shown in Figures 4A–D. In T2DM group, the time spent in TMT-B was negatively correlated with FA (*R* = −0.377, *P* = 0.037), volume (*R* = −0.446, *P* = 0.012), and surface area (*R* = −0.493, *P* = 0.005) of the reconstructed tract. Noteworthy is that the FA, volume, and surface area were significantly correlated with each other (all *P*-values < 0.01). Due to the significant effects of disease duration on the CFT-Delay trial, we further included disease duration as a covariate in the partial correlation analyses. However, the performance was not correlated with any of the diffusion measures. Among all the endocrine variables, IR was the only one that correlated with the diffusion measures of tract length (*R* = −0.436, *P* = 0.042).

## Discussion

In the current study, we characterized the microstructure of bilateral CB and its relationship with cognitive performance and endocrine measurements. Using fiber tractography, we demonstrated reduced FA, increased MD, shorter fibers, and a trend of the smaller tract in the CB in diabetic patients, which were independent of WM lesions. Most importantly, the impairments in the CB were significantly correlated with the worse performance in the TMT-B and elevated IR in the patients’ group.

Several existing studies using different modes of DTI analyses have consistently reported diffusion abnormalities in the CB in T2DM patients (Sanjari Moghaddam et al., 2019). Most of them have surveyed the whole brain relying on either VBA or TBSS method, providing results limited to regional clusters instead of treating the tract as a whole. The only study known to us that used the tractography method was based on manually drawn ROIs, which would be prone to anatomical misplacement (Hoogenboom et al., 2014). In comparison, the atlas-guided automatic tract reconstruction in the present study enabled us to clearly depict the CB in its entirety. Without the need for extensive anatomical experience and high reproducibility of ROI placement, such an approach could minimize false-positive tracing and prominently improve the stability of the diffusion measurements (Reich et al., 2010). Taken together, we consider our results to be more CB-specific and reproducible than the existing literature.

Our results indicated that both FA and MD are significantly differed between the two groups, especially when there was little overlay between these abnormalities and the WMH revealed by conventional MRI. These results suggested that the cingulum abnormalities were independent of WMH, while DTI is clearly complementary to traditional MRI in detecting subtle WM abnormalities. As a core part of the limbic system (Dalgleish, 2004), CB has complex connections and multiple functions, being consistently reported to be injured in a wide range of neurological and psychiatric disorders. The disruption of CB in T2DM is thus not unexpected, which might reflect an overlapping pattern during the process of cognitive decline. Besides, we and several other studies have previously reported DMN disconnection in T2DM patients, especially in its posterior components (Cui et al., 2015; Ishibashi et al., 2018; Liu et al., 2019). Given that CB is the anatomical basis for DMN (van den Heuvel et al., 2008), the present CB abnormalities not only are supported by the previous functional results but also extend fMRI studies by adding anatomical evidence. Nonetheless, our results revealed no difference in the fiber number, volume, or surface area, which might be attributed to the large individual variance, as shown by the SD values in the results.

The disrupted CB may be associated with pathologic processes of T2DM. As two representative diffusion metrics, FA indicates the degree of anisotropic movement of water molecules, while MD measures the average movement in three possible dimensional directions (Alexander et al., 2007). Briefly, the reduced FA and increased MD, as shown in our results, are indicative of lower microstructural integrity within the CB. Similarly, lower FA and higher MD values have been frequently reported in T2DM patients in diffuse WM tracts, although the regions tend to differ across studies (Hoogenboom et al., 2014; Raffield et al., 2016; Yoon et al., 2017). Diabetes is often associated with an increased burden of SVD, leading to WM impairment that is sensitive and susceptible to ischemic stress (Hardigan et al., 2016). A recent clinical study also suggested diabetes as an important risk factor for WMH progression (Tamura and Araki, 2015). Although the changes in diffusion parameters might reflect myelin or axon pathology according to their physiological nature (Alexander et al., 2007), the specific neuropathology remains unproved and merits further exploration.

VBA suggested that the disruption has a predilection for the posterior CB segment. According to anatomical reviews, the CB can be at least divided into the dorsal and ventral components, each having distinct connections and functions, although how best to subdivide the cingulum is still under debate (Bubb et al., 2018). The present cluster with significant group difference lies in the posterior part of the dorsal CB, i.e., the “retrosplenial” component, which is closest anatomically to the posterior cingulate cortex (PCC) and connects the medial frontal and parietal lobes along the cingulate cortices (Jones et al., 2013). As core components of the well-defined DMN, the dysfunction of PCC has been observed in various neurodegenerative conditions and is considered hallmarks of cognitive decline (Greicius et al., 2004). Similarly, both volumetric, metabolic, and perfusion reduction in the PCC region have been reported in T2DM patients (Baker et al., 2011; Cui et al., 2017). Given the close anatomical connections, it is not unexpected to detect the abnormalities in the retrosplenial component. Interestingly, reduced FA in posterior CB is a consistent finding in MCI and AD (Metzler-Baddeley et al., 2012), in contrast to normal aging that mainly involves the anterior component (Sibilia et al., 2017). T2DM is an established risk factor for developing all-cause dementia, yet the link is mediated through vascular dysfunction, AD neuropathologic changes, or both, remain debated (Pruzin et al., 2018). Although our results suggested comparable CB abnormalities in T2DM and AD patients, whether T2DM is more likely to develop AD pathologies needs more evidence.

Several diffusion measures of the reconstructed tract that pass through the group-differed cluster were exclusively correlated with the performance in TMT-B. As mentioned above, the tract belongs to the retrosplenial segment, the integrity of which is associated with not only memory but also visuospatial processing and executive functions (Vogt et al., 1995), which covered the main cognitive domains that are required in the successful performance of the TMT-B (Chen et al., 2009). Therefore, the decline in executive functioning, which is one of the key manifestations in T2DM-related cognitive impairment, might be attributed to the disruption in CB integrity. On the other hand, IR rather than the glucose level was the only endocrine parameters correlated with the diffusion measures. IR, the central pathological feature of T2DM, is hypothesized to interfere with normal synaptic transmission and plasticity, which may be implicated as part of the pathogenic process of dementia (Ferrario and Reagan, 2018). Based on our results, we speculated that IR might be an important risk factor for developing T2DM-related cognitive impairment. The insignificant correlation between glucose level and DTI measurements might be interpreted as the glucose fluctuations and the effects of various medications.

Several limitations of our study need to be addressed. First, the small sample size may have limited the detection of group differences and our interpretation of the current results. Second, the current results might be confounded by the presence of T2DM-related complications and comorbidities. However, we did control for the possible risk factors, which should have minimized the confounding effects. Third, most of our patients and some HC were taking various medications. Further studies should include more participants and to test the effects of interventions. Finally, there are deficits in the specificity and precision of the DTI metrics, especially when the targeted areas have complex crossing fibers. More advanced imaging methodologies such as diffusion spectrum and kurtosis imaging should be applied, and more WM tracts should be investigated using multi-modality design.

Our findings in T2DM patients showed significant alterations in bilateral CB, while the most prominent difference was found in the right retrosplenial segment. More importantly, the diffusion metrics of the reconstructed tract passing the group-differed cluster were significantly correlated with cognitive performance and IR level. These results not only addressed the role of disrupted major WM tract in the process of T2DM-related cognitive decline but also highlighted IR as an important risk factor. However, the underlying pathophysiology of these microstructural changes should be examined in future studies.

## Data Availability Statement

The datasets generated for this study are available on request to the corresponding author.

## Ethics Statement

The studies involving human participants were reviewed and approved by IEC for Clinical Research, Zhongda Hospital, Southeast University. The patients/participants provided their written informed consent to participate in this study.

## Author Contributions

SJ conceived and designed the experiments. YCu and T-YT collected and analyzed the MR data, and wrote, reviewed, and edited the manuscript. C-QL, YCa, and TL contributed to data quality control and data analyses. Y-CW and G-JT reviewed and edited the manuscript. All authors contributed to the article and approved the submitted version.

## Conflict of Interest

The authors declare that the research was conducted in the absence of any commercial or financial relationships that could be construed as a potential conflict of interest.

## Footnotes

^1^www.statistical-modelling.de/lst.html

^2^http://dsi-studio.labsolver.org/

^3^http://www.fmrib.ox.ac.uk/fsl/

^4^https://pitt.app.box.com/v/HCP1021-1mm
